# Neuroinflammation and Brain Disease

**DOI:** 10.1186/s12883-023-03252-0

**Published:** 2023-06-12

**Authors:** A. Bersano, J. Engele, M.K.E. Schäfer

**Affiliations:** 1 Cerebrovascular Unit, Fondazione IRCCS Istituto Neurologico Carlo Besta Milan, Via Celoria 11, Milan, 20133 Italy; 2 Institute of Anatomy, Leipzig University, Leipzig, Germany; 3 Department of Anesthesiology, University Medical Center of the Johannes Gutenberg- University, Mainz, Germany

## Abstract

Starting from the perspective of an immune-privileged site, our knowledge of the inflammatory processes within the central nervous system has increased rapidly over the last 30 years, leading to a rather puzzling picture today. Of particular interest is the emergence of disease- and injury-specific inflammatory responses within the brain, which may form the basis for future therapeutic approaches. To advance this important topic, we invite authors to contribute research and clinical papers to the Collection “Neuroinflammation and Brain Disease”.

Inflammation is a biological process that dynamically alters the surrounding microenvironment, including participating immune cells [1]. Surrounded by specialized barriers and with immune-specific properties, the central nervous system (CNS) tightly regulates immune responses [2]. In ‘neuroinflammatory’ conditions, pathogenic immunity can disrupt CNS structure and function [3]. Neuroinflammation has been observed as a key pathway in the onset and/or progression of several neurological disorders defined as inflammatory (e.g., multiple sclerosis, vasculitis, etc.), but also in neurological conditions not usually categorized as inflammatory, such as Alzheimer’s disease (AD), Parkinson’s disease, amyotrophic lateral sclerosis, stroke and traumatic brain injuries (TBI) [4–8].

The activation of glial cells and complement-mediated pathways, the synthesis of inflammation mediators, and the recruitment of leukocytes, are key elements of brain inflammation. Under the influence of exogenous and endogenous factors (e.g., trauma, stroke, chronic infections, disease-related proteins like amyloid-β (Aβ), tau/p-tau or α-synuclein), the activation of microglia triggers several signal transduction pathways, including phosphoinositide 3-kinase/protein kinase B (PI3K/AKT), mitogen-activated protein kinase (MAPK) and mammalian target of rapamycin (mTOR), leading to transcription factor nuclear factor kappa-light-chain-enhancer of activated B cells (NF-κB) activation (9–10). The subsequent production of pro-inflammatory cytokines, chemokines, inducible enzymes (e.g., inducible nitric oxide synthase -iNOS) and cyclooxygenase 2 (COX-2) drive neuroinflammation. Numerous studies have indeed documented the increased production of different cytokines, including interleukin-1β (IL-1β), IL-6, IL-18, IL-12, IL-23, IL-33 and tumor necrosis factor-α (TNF-α), in various neurological and neuropsychiatric disorders [11]. For example, high expression of IL-1β in microglia cells surrounding Aβ plaques was observed in AD patients [12]. Moreover, the neuroinflammation observed in neurological disorders has a pivotal role in exacerbating Aβ burden and tau hyperphosphorylation, suggesting that stimulating cytokines in response to an undesirable external response could be a checkpoint for treating neurological disorders.

It has become clear that inflammation also contributes to pathological, clinical and functional outcomes in the context of acquired brain injuries such as TBI and stroke [7]. It is noteworthy that acquired brain injuries represent a risk factor for the chronic neurodegenerative diseases mentioned above. Much research has focused on the role of brain-resident microglia, the primary immune cells in the CNS, and astrocytes, and how they either exacerbate inflammatory damage or help to maintain a healthy environment in the CNS. However, the duality of inflammatory reactions, often referred to as the “double-edged sword”, is still challenging and complicates the development of therapeutic options [13, 14]. The underlying mechanisms of neuroinflammation are likely to involve multiple cell types and knowledge about their in vivo interactions remains elusive. This not only applies to brain-resident cells such as neurons, astrocytes, microglia, oligodendrocytes and neural progenitor cells, but also to the role of early infiltrating and possibly persisting peripheral immune cells, such as monocytes, macrophages, neutrophils, and T cells. Therefore, it is necessary to decipher the crosstalk between various cell types, identify differences and commonalities in molecular signaling pathways, and modulate critical signaling pathways, in order to gain a more complete knowledge to develop therapeutic strategies for treatment. This could become possible through the integration of network modeling approaches for multi-omics at the tissue and single-cell level (15–16).

Another level of complexity arises from crosstalk between the brain and other organs. Several studies have reported on reciprocal interactions between the injured or diseased brain with the gut microbiome and how therapeutic drugs may influence these interactions [17–20]. Moreover, organ dysfunction has been recognized to be bidirectional, meaning that dysfunction in one organ potentiates injury to others. Scientists are just beginning to understand how these processes trigger neuroinflammation. For example, TBI can negatively impact various organs, including the pulmonary, gastrointestinal, cardiovascular, renal, and immune systems [21]. Furthermore, it should also be considered that sex, age and comorbidities can strongly influence inflammatory responses in acute and chronic neurodegeneration (22–23). Finally, to translate results from bench to bedside, consistent improvement and application of diagnostic and prognostic tools, including functional neuroimaging, advanced magnetic resonance imaging processing and meaningful biomarkers [24] to characterize the timing, localization, extent, and duration of inflammation are clearly important. The identification of suitable biomarkers could be promoted, for example, by unified classification schemes to assess their clinical utility [25–26]. A better understanding of the role that inflammatory processes play in the natural history of diseases is essential to identify potential therapeutic targets and develop integrated pharmacological approaches acting at different levels and stages of disease. We hope that this collection will provide a useful platform for articles that address focused research questions on molecular and cellular mechanisms in the area of neuroinflammation and brain diseases, and also provide ideas for integrative organism-level approaches and perspectives on therapeutic options.

## Data Availability

N/A.

## Abbreviations

CNS: Central nervous system
TBI: Traumatic brain injury
Aβ: Amyloid-β
PI3K/AKT: Phosphoinositide 3-kinase/protein kinase B
MAPK: Mitogen-activated protein kinase
mTOR: Mammalian target of rapamycin
NF-κB: Nuclear factor kappa-light-chain-enhancer of activated B cells
iNOS: Inducible nitric oxide synthase
COX-2: Cyclooxygenase 2
IL-1β: Interleukin-1β
IL-6: Interleukin-6
IL-18: Interleukin-18
IL-12: Interleukin-12
IL-23: Interleukin-23
IL-33: Interleukin-33
TNF-α: Tumor necrosis factor-α
AD: Alzheimer's disease
